# Long-Acting Injectable Cabotegravir for HIV Preexposure Prophylaxis Among Sexual and Gender Minorities: Protocol for an Implementation Study

**DOI:** 10.2196/44961

**Published:** 2023-04-19

**Authors:** Beatriz Grinsztejn, Thiago Silva Torres, Brenda Hoagland, Emilia Moreira Jalil, Ronaldo Ismerio Moreira, Gabrielle O'Malley, Starley B Shade, Marcos R Benedetti, Julio Moreira, Keila Simpson, Maria Cristina Pimenta, Valdiléa Gonçalves Veloso, The ImPrEP CAB-Brasil Study Team

**Affiliations:** 1 Instituto Nacional de Infectologia Evandro Chagas, Fundação Oswaldo Cruz Rio de Janeiro Brazil; 2 Department of Global Health Schools of Medicine and Public Health University of Washington Seattle, WA United States; 3 Institute for Global Health Sciences, Department of Epidemiology and Biostatistics University of California San Francisco San Francisco, CA United States; 4 Grupo Arco-Íris de Cidadania LGBT Rio de Janeiro Brazil; 5 Associação Nacional de Travestis e Transexuais Salvador Brazil; 6 Brazilian Ministry of Health Brasilia Brazil; 7 See Acknowledgments

**Keywords:** sexual and gender minorities, young, Brazil, HIV prevention, injectable preexposure prophylaxis, injectable PrEP, cabotegravir

## Abstract

**Background:**

Long-acting injectable cabotegravir (CAB-LA) for preexposure prophylaxis (PrEP) has proven efficacious in randomized controlled trials. Further research is critical to evaluate its effectiveness in real-world settings and identify effective implementation approaches, especially among young sexual and gender minorities (SGMs).

**Objective:**

ImPrEP CAB Brasil is an implementation study aiming to generate critical evidence on the feasibility, acceptability, and effectiveness of incorporating CAB-LA into the existing public health oral PrEP services in 6 Brazilian cities. It will also evaluate a mobile health (mHealth) education and decision support tool, digital injection appointment reminders, and the facilitators of and barriers to integrating CAB-LA into the existing services.

**Methods:**

This type-2 hybrid implementation-effectiveness study includes formative work, qualitative assessments, and clinical steps 1 to 4. For formative work, we will use participatory design methods to develop an initial CAB-LA implementation package and process mapping at each site to facilitate optimal client flow. SGMs aged 18 to 30 years arriving at a study clinic interested in PrEP (naive) will be invited for step 1. Individuals who tested HIV negative will receive mHealth intervention and standard of care (SOC) counseling or SOC for PrEP choice (oral or CAB-LA). Participants interested in CAB-LA will be invited for step 2, and those with undetectable HIV viral load will receive same-day CAB-LA injection and will be randomized to receive digital appointment reminders or SOC. Clinical appointments and CAB-LA injection are scheduled after 1 month and every 2 months thereafter (25-month follow-up). Participants will be invited to a 1-year follow-up to step 3 if they decide to change to oral PrEP or discontinue CAB-LA and to step 4 if diagnosed with HIV during the study. Outcomes of interest include PrEP acceptability, choice, effectiveness, implementation, and feasibility. HIV incidence in the CAB-LA cohort (n=1200) will be compared with that in a similar oral PrEP cohort from the public health system. The effectiveness of the mHealth and digital interventions will be assessed using interrupted time series analysis and logistic mixed models, respectively.

**Results:**

During the third and fourth quarters of 2022, we obtained regulatory approvals; programmed data entry and management systems; trained sites; and performed community consultancy and formative work. Study enrollment is programmed for the second quarter of 2023.

**Conclusions:**

ImPrEP CAB Brasil is the first study to evaluate CAB-LA PrEP implementation in Latin America, one of the regions where PrEP scale-up is most needed. This study will be fundamental to designing programmatic strategies for implementing and scaling up feasible, equitable, cost-effective, sustainable, and comprehensive alternatives for PrEP programs. It will also contribute to maximizing the impact of a public health approach to reducing HIV incidence among SGMs in Brazil and other countries in the Global South.

**Trial Registration:**

Clinicaltrials.gov NCT05515770; https://clinicaltrials.gov/ct2/show/NCT05515770

**International Registered Report Identifier (IRRID):**

PRR1-10.2196/44961

## Introduction

### Background

In 2021, there were approximately 960,000 people living with HIV in Brazil [1]. In recent studies, the HIV prevalence rate was estimated to reach approximately 23% among Brazilian gay men, bisexual men, and other men who have sex with men (MSM) [2] and surpass 30% among *travesti* and transgender women (TGW) in some cities [3,4]. Young MSM aged ≤30 years are among the few populations with an increasing number of new HIV infections in Brazil, whereas HIV incidence has stabilized in the overall population [2]. Since December 2017, oral tenofovir disoproxil fumarate (TDF) and emtricitabine (FTC) HIV preexposure prophylaxis (PrEP) have been freely available in Brazil through the national public health system (*Sistema Único de Saúde* [SUS]) [5]. As of October 2022, there were 566 health facilities across all Brazilian states providing oral PrEP to approximately 46,886 individuals [6]. Although 85% of current PrEP users in Brazil are MSM, PrEP use remains well below the initial projections [5].

Tenofovir-based oral PrEP has been effective in dramatically reducing population-level HIV incidence in multiple regions [7-11]. However, to realize the protective benefits of PrEP, individuals need to both initiate PrEP and remain on PrEP during periods of HIV vulnerability [12,13]. Among the individuals who initiated PrEP in the Brazilian SUS, discontinuation was common among MSM (approximately 40%) and TGW (>50%) [6]. PrEP Brasil, a daily oral PrEP demonstration study, found that although 74% of all participants had protective concentrations of tenofovir diphosphate at week 48, young participants and TGW participants had decreasing rates of protective drug concentrations throughout the study [14]. In addition, data from the ImPrEP study, which aimed to evaluate the feasibility of same-day PrEP initiation for MSM and TGW within the public health contexts of Brazil, Mexico, and Peru, identified lower PrEP adherence among TGW and individuals aged ≤30 years [15,16].

Owing to user adherence challenges, PrEP has not decreased HIV incidence among some population, particularly among populations with higher susceptibility (youth, racial and ethnic minorities, and TGW) [17-20]. Although HIV incidence in the ImPrEP study population was relatively low (0.85/100 person-years), it was higher among TGW, participants from Peru, those aged 18 to 30 years, Black or mixed-race participants, and those who were nonadherent to daily oral PrEP [15,16]. These disproportionately worse outcomes reinforce the potential of PrEP agents that do not require daily or planned oral dosing to increase their effective use among the most susceptible populations.

In multiple studies, reasons for discontinuing or never initiating daily oral PrEP include low HIV perceived risk, concerns about medication side effects, desire not to take a daily pill, stigma, competing life events, and challenges accessing the medication [13,21-23]. Long-acting PrEP agents may potentially address some of these concerns by providing consistent protection through infrequent, discrete administration, avoiding the need for daily adherence to an oral regimen and the related need for disclosure to sex partners and acquaintances [24].

### Long-Acting Injectable Cabotegravir

Long-acting injectable cabotegravir (CAB-LA) is an integrase strand-transfer inhibitor, and its effectiveness for PrEP has been demonstrated in phase 2b/3 studies (HIV Prevention Trials Network [HPTN] 083 and HPTN 084) [25,26]. HPTN 083 was a double-blind double-dummy randomized controlled trial that assessed the effectiveness of CAB-LA for PrEP compared with oral tenofovir disoproxil fumarate (TDF) and emtricitabine (FTC) PrEP among 4566 cisgender MSM and TGW under high susceptibility to HIV. The blinded phase of the study showed the superiority of CAB-LA, with a 66% reduction in new infections in the cabotegravir arm compared with the active TDF and FTC arm. Similarly, HPTN 084 observed impressive reductions in HIV infections in the CAB-LA arm compared with the TDF and FTC arm among cisgender women in Africa, with an 89% reduction in new infections in the cabotegravir arm when compared with the active TDF and FTC arm. Data from both studies led to the regulatory approval of CAB-LA for HIV prevention in the United States, Australia, South Africa, Malawi, and Zimbabwe. Approval is currently being sought in numerous countries, including Brazil. In July 2022, a recommendation for CAB-LA PrEP was issued by the World Health Organization (WHO) [27].

Although it is reassuring that CAB-LA has demonstrated comparable effectiveness among youth, Black MSM, and TGW in large clinical trials, the advantages of bypassing daily adherence behavior may not be realized if individuals cannot access injectable PrEP or do not return for repeat injections. Implementation studies are needed to provide crucial information about how interventions that are proven efficacious under randomized clinical trial conditions can be optimized for delivery within real-world service settings. The ImPrEP CAB Brasil implementation study aims to generate critical evidence to inform national policies and program implementers about how injectable CAB-LA PrEP can be provided to MSM, nonbinary persons, and transgender persons within public health PrEP services in Brazil. Its implementation objectives are to describe the facilitators of and barriers to integrating CAB-LA into routine public health clinics and to evaluate whether a mobile health (mHealth) intervention optimizes decision-making on the best PrEP regimen among PrEP users and WhatsApp text messaging improves the uptake of and adherence to CAB-LA injection appointments. The primary clinical objective is to evaluate the effectiveness of CAB-LA PrEP in reducing the risk of HIV acquisition in a study cohort of participants in a context where participants can exercise choice in their HIV prevention method (injectable CAB-LA PrEP or daily oral PrEP).

## Methods

### Study Design

**Figure 1:**
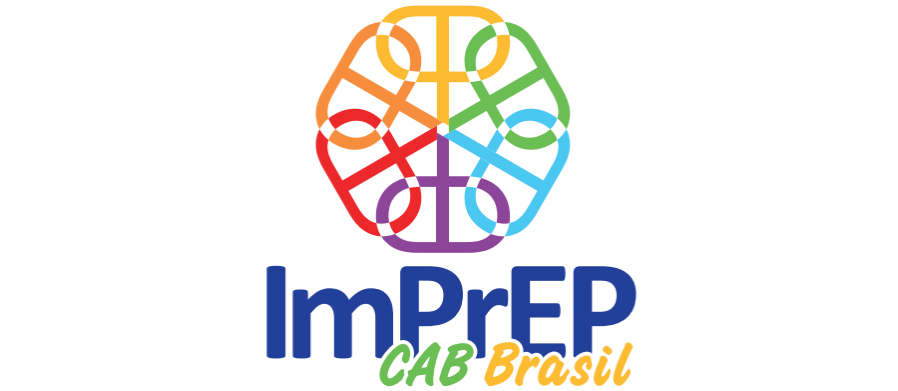
ImPrEP CAB Brasil logo.

**Figure 2:**
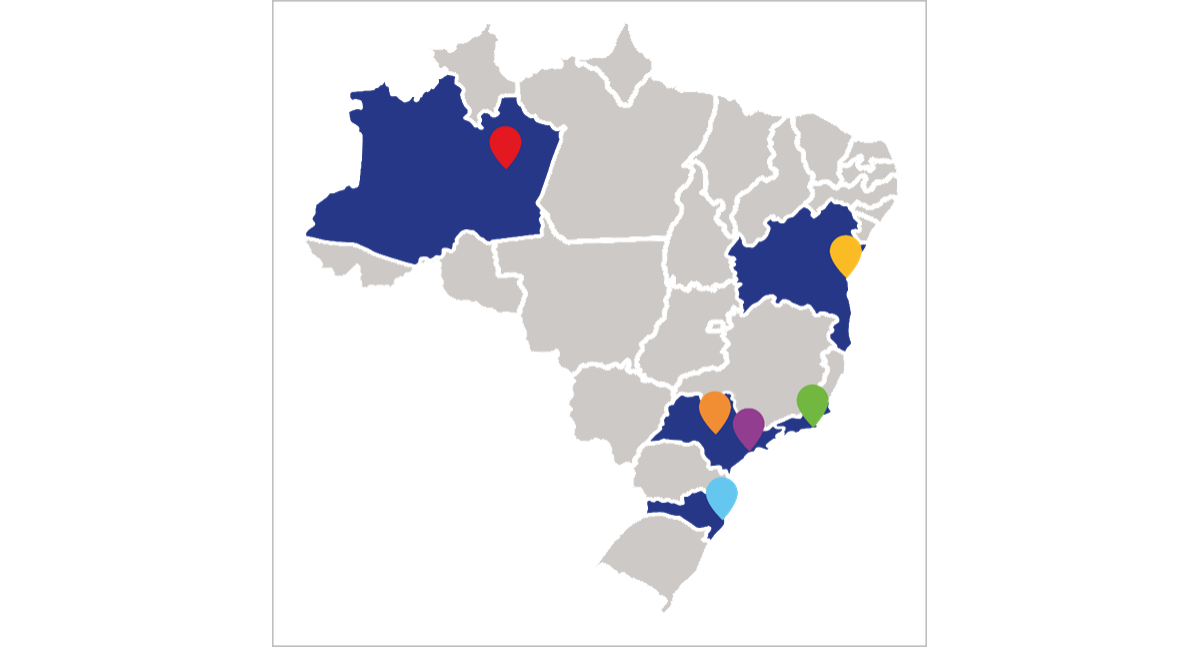
Site distribution for the ImPrEP CAB Brasil study.

The ImPrEP CAB Brasil (Figure 1) is an implementation study of CAB-LA PrEP among 1200 young adult (aged 18 to 30 years) sexual and gender minorities (SGMs) in 6 Brazilian cities: Rio de Janeiro, São Paulo, Manaus, Salvador, Florianópolis, and Campinas (Figure 2). Each participating site provides PrEP service as a part of the Brazilian SUS.

This is a type-2 hybrid implementation-effectiveness open-label cohort study with a convergent mixed methods approach (quantitative and qualitative). A type-2 hybrid design examines both effectiveness and implementation strategies within the same study [28]. The study will draw on selected implementation science constructs from the theoretical framework for acceptability (TFA) [29], the Reach, Effectiveness, Adoption, Implementation, and Maintenance framework [30], and the framework of Proctor et al [31] to assess the implementation outcomes for the different study objectives outlined earlier. Relevant outcomes and their level of evaluation will include the following: acceptability at the individual health provider and participant levels, reach (choice and uptake) at the individual participant level, effectiveness at the individual participant level, implementation at the setting level (overall delivery), maintenance at the individual participant level, and feasibility at the setting level.

**Figure 3:**
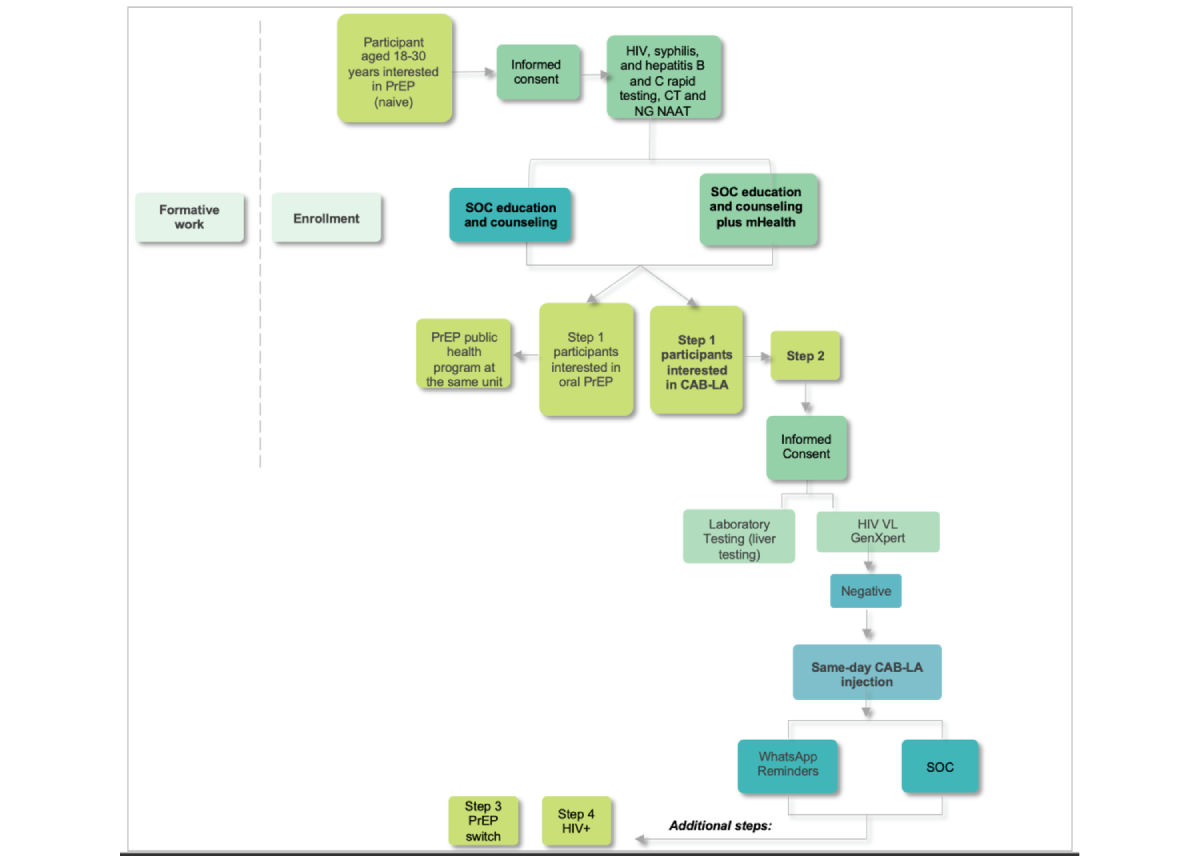
Overview of the study design and enrollment scheme. Only HIV-negative participants will perform standard of care (SOC) or mobile health (mHealth). Individuals living with HIV will be referred for antiretroviral therapy. CAB-LA: long-acting injectable cabotegravir; CT: Chlamydia trachomatis; NAAT: nucleic acid amplification testing; NG: Neisseria gonorrhea; PrEP: preexposure prophylaxis; VL: viral load.

Individuals may participate in up to 4 different steps (Figure 3). In step 1, PrEP naive individuals who come to the study sites seeking PrEP will be exposed to education and counseling about HIV prevention and be informed of the availability of both oral and injectable PrEP. Half of the enrolled participants that tested negative for HIV will receive standard of care (SOC) prevention counseling, and the other half will be exposed to an mHealth education and decision support intervention in addition to SOC prevention counseling. Participants who choose to use injectable PrEP and with an undetectable HIV viral load will enroll in step 2 on the same day, whereas those who choose to use oral PrEP will be referred to the Brazilian Public Health PrEP program at the same clinic. Of the participants who enroll in step 2 and receive a CAB-LA injection, half will be randomized to receive a digital appointment reminder using WhatsApp (Meta Platforms, Inc; the most common messaging app in Brazil). Participants in step 2 who may decide to discontinue CAB-LA during the study follow-up will be invited to step 3 and switch to oral PrEP (TDF and FTC) or keep off PrEP with HIV and sexually transmitted infection (STI) monitoring and counseling for 1 year. Participants from either step 2 or step 3 who are diagnosed with an HIV infection during follow-up will be invited to be enrolled in step 4, where antiretroviral therapy (ART) effectiveness and ART resistance will be monitored for 1 year.

### Eligibility Criteria

Participants will be included if they (1) are MSM, nonbinary person (assigned as male at birth), TGW, or transgender men; (2) are aged 18 to 30 years; (3) attend one of the study clinics looking for PrEP; (4) are CAB-LA and TDF and FTC PrEP naive; (5) are willing and able to provide written informed consent and adhere to the study requirements; (6) have nonreactive or negative HIV test results, including both third-generation HIV rapid tests and an undetectable HIV RNA at enrollment for individuals choosing injectable CAB-LA; (7) have no report of hepatic dysfunction; and (8) are willing to undergo all required study procedures. The full list of exclusion criteria is described in Multimedia Appendix 1. The study team will provide checklists to aid the site teams in accessing the inclusion and exclusion criteria.

### Study Procedures

#### Formative Work

We will conduct formative work using participatory design methods [32] to develop an initial implementation package for CAB-LA PrEP. Training modules will be developed in collaboration with health providers at the 2 sites that have had experience with CAB-LA PrEP as part of HPTN 083 (Rio de Janeiro and São Paulo) and will include slides, learning activities, and job aids (eg, education, and counseling scripts).

Process mapping [33,34] at each site will facilitate planning for optimal PrEP client flow, that is, for ensuring that PrEP users can move most efficiently and effectively through the various clinical steps needed to receive services while minimizing the work burden on health care providers and maintaining service quality. Engaging clinical staff in the process mapping exercise prompts the identification of anticipated facilitators and barriers associated with each step of service delivery as well as planning for barrier mitigation strategies. Thus, process maps can facilitate the development of “readiness” exercises once PrEP CAB-LA is approved and ready for scale-up in Brazil and elsewhere. Finally, process maps will also be used at the end of the study to guide health care providers’ reflections on the facilitators of and barriers to different components of PrEP delivery. Guidelines for developing process maps and process map examples will be included in the implementation package. The implementation package developed in collaboration with providers from the CAB-LA–experienced sites will be used to prepare health care providers at the remaining 4 sites and will be revised based on their feedback.

Focus group discussions (FGDs) will be held with health care providers experienced in CAB-LA PrEP and members of the young SGM community to gather their input for the adaptation of an mHealth education and decision support tool. This tool, intended to be accessed via an electronic tablet with self-guided instructions, will be designed to convey information about both oral and CAB-LA PrEP in an engaging way, describing the benefits and drawbacks of each method, to help participants decide whether and which PrEP method may be appropriate for them. A preliminary storyboard will be developed and shared with FGD participants, and they will be asked to provide feedback on images, content, and sequence. Feedback will be incorporated into the programming of the mHealth education intervention. FGD participants will be also asked to review and refine the content of WhatsApp appointment reminders and provide input regarding the best timing for receiving the WhatsApp reminders. In addition, FGD participants will be asked about barriers and facilitators for CAB-LA PrEP implementation. Health care providers at the 2 study sites with experience in CAB-LA PrEP will be inquired about frequent questions asked by CAB-LA PrEP users about the medication as well as examples of education and counseling strategies they have used previously to facilitate CAB-LA user comprehension.

#### Step 1: mHealth Intervention

##### Overview

Step 1 focuses on evaluating the mHealth intervention and participants’ choice of PrEP method (Table 1). Potential step 1 participants include individuals who come to a study clinic looking for PrEP, are PrEP naive, and have expressed an interest in taking PrEP (see eligibility criteria). All individuals accessing the service for PrEP will be assessed for study eligibility by a designated study staff member. Eligible participants will be asked whether they are interested to assess educational and counseling interventions for PrEP choice.

After signing step 1 informed consent, participants will undergo rapid tests for HIV, syphilis, and hepatitis B and C. Rectal, oral, and urine swab samples will be collected for *Chlamydia trachomatis* and *Neisseria gonorrhoeae* nucleic acid amplification testing. If a reactive or positive result is obtained for HIV test, the respective person is deemed ineligible to receive PrEP. Additional testing to confirm suspected HIV infection will be performed in accordance with local guidelines. If an HIV infection is confirmed, the participants will receive counseling and be immediately referred for HIV treatment and care. All participants will be evaluated for signs or symptoms of a possible acute HIV infection. Those with suspected acute HIV infection will be referred for follow-up investigation within the public health system.

Participants at each site will receive counseling and education on the use of PrEP options per the local SOC from a health care provider until 100 participants indicate that they would like to start CAB-LA PrEP and enroll in step 2. Additional participants will receive an mHealth education and counseling intervention plus the SOC education and counseling until 100 participants exposed to both SOC and the mHealth intervention enroll in step 2 of the study at each site (200 participants enrolled per site).

Upon completing their exposure to SOC or SOC plus mHealth, participants will be asked to complete a brief survey administered by a study member that will capture the following information: (1) participants’ knowledge and beliefs about the dosing, advantages, and disadvantages of the 2 PrEP options, (2) their method of choice for HIV prevention, and (3) the reasons for their preference for oral or CAB-LA PrEP. Participants who received the mHealth intervention will be asked additional questions about its acceptability and its value in supporting decision making on PrEP choice.

**Table 1:** Overview of the ImPrEP CAB Brasil study visits and schedule of procedures: steps 1 and 2 (year 1).

Procedures		Step 1 enrollment	Step 2								
			Enrollment	Month 1	Month 3	Month 5		Month 7	Month 9	Month 11	Month 13 or year 1
**Administrative, behavioral, and clinical**											
	ICF^a^ step 1	✓									
	SOC^b^ vs mHealth^c^ and SOC	✓									
	Health provider survey	✓									
	PrEP^d^ choice	✓									
	ICF step 2		✓								
	Randomization text message		✓								
	Locator information		✓	✓	✓	✓		✓	✓	✓	✓
	HIV counseling		✓	✓	✓	✓		✓	✓	✓	✓
	Reproductive counseling^e^		✓	✓	✓	✓		✓	✓	✓	✓
	Condom, lubricant, and HIVST^f^		✓	✓	✓	✓		✓	✓	✓	✓
	Adherence counseling		✓	✓	✓	✓		✓	✓	✓	✓
	HIV risk and mental health assessment		✓	✓	✓	✓		✓	✓	✓	✓
	Medical history		✓	✓	✓	✓		✓	✓	✓	✓
	Concomitant medication		✓	✓	✓	✓		✓	✓	✓	✓
	Directed physical examination		✓	✓	✓	✓		✓	✓	✓	✓
	Weight and vital signs		✓	✓	✓	✓		✓	✓	✓	✓
	Height		✓								✓
	HIVST report			✓	✓	✓		✓	✓	✓	✓
**Laboratory**											
	Third-generation HIV rapid test	✓		✓	✓	✓		✓	✓	✓	✓
	HIV RNA PCR^g^ (GeneXpert)		✓								✓^h^
	HBV^i^ rapid test	✓			✓			✓		✓	✓
	HCV^j^ rapid test	✓			✓			✓		✓	✓
	Syphilis test^k^	✓			✓			✓		✓	✓^h^
	Liver function test (ALT^l^)		✓		✓			✓		✓	✓
	Oropharyngeal CT^m^ and NG^n^ test	✓			✓			✓		✓	✓^h^
	Urine CT and NG test	✓			✓			✓		✓	✓^h^
	Rectal swab CT and NG test	✓			✓			✓		✓	✓^h^
	Urine pregnancy test		✓^e^	✓^e^	✓^e^	✓^e^		✓^e^	✓^e^	✓^e^	✓^e^
	Plasma and Serum storage		✓	✓	✓	✓		✓	✓	✓	✓
**Study medication**											
	Injection		✓	✓	✓	✓		✓	✓	✓	✓
	ISR^o^ evaluation		✓	✓	✓	✓		✓	✓	✓	✓

^a^ICF: informed consent form.

^b^SOC: standard of care.

^c^mHealth: mobile health.

^d^PrEP: preexposure prophylaxis.

^e^Only for transgender men.

^f^HIVST: HIV self-testing.

^g^PCR: polymerase chain reaction.

^h^Collect if it is the final study visit.

^i^HBV: hepatitis B.

^j^HCV: hepatitis C.

^k^Rapid test plus nontreponemal syphilis testing, as indicated.

^l^ALT: alanine aminotransferase.

^m^CT: *Chlamydia trachomatis*.

^n^NG: *Neisseria gonorrhea*.

^o^ISR: injection site reaction.

##### Qualitative Interview

A small subset of step 1 participants from each site (8 SOC participants per site and 12 SOC plus mHealth participants per site) will be asked to participate in a qualitative interview to evaluate their perceived advantages and disadvantages of different PrEP products and reasons for their choice. Those who received SOC plus mHealth will be asked to share their feedback on the acceptability of the mHealth intervention. Audio recordings will be transcribed *verbatim* by study staff.

##### PrEP Choice and Administration

Following the completion of step 1 procedures, participants will be asked by a health provider whether they choose oral PrEP or CAB-LA PrEP. Participants who indicate a preference for oral PrEP will be referred to receive it in the routine HIV prevention service and will not be navigated by the study team. The study team will not actively follow-up on these participants but will retain their participant ID in case they express interest in switching to CAB-LA PrEP later in the study period. Participants who choose CAB-LA PrEP will be invited on the same day to participate in step 2 of the study and will undergo additional consent, screening, HIV viral load testing, enrollment, and data collection procedures.

#### Step 2

##### Overview

Step 1 participants who chose CAB-LA PrEP after receiving either SOC counseling or SOC counseling plus mHealth will be eligible to participate on the same day in step 2 (Tables 1 and 2). Step 2 aims to understand the safety and effectiveness of CAB-LA PrEP in an environment of choice, assess an implementation strategy (WhatsApp appointment reminder) for improving adherence to CAB-LA injections, evaluate HIV testing strategies, evaluate the acceptability of STI sample self-collection and HIV self-testing, and understand the facilitators of and barriers to the integrated delivery of CAB-LA PrEP in the existing oral PrEP services.

HIV viral load testing will be performed in step 2 participants using the GeneXpert platform to confirm their eligibility. With a detectable HIV result, the person will be deemed ineligible to receive PrEP and will be referred to treatment and care through the public health system. Those who do not have detectable HIV results and do not meet any other exclusion criteria will receive their first CAB-LA injection.

The first follow-up visit will happen in 1 month, and the subsequent follow-up visits will happen once in every 2 months. At every follow-up visit, sites will perform HIV testing using the third-generation (antibody) HIV rapid tests before providing the next CAB-LA injection. Blood-based HIV self-testing will be performed by the study participant 24 hours before all follow-up visits, and the results will be reported by the participant during the study visit. If a participant does not bring the HIV self-testing report, the test will be performed at the clinic in a private space and before the study visit procedures. Regularly, additional HIV testing with stored samples will be conducted retrospectively, in batches, with fourth-generation HIV rapid test, Abbot ARCHITECT HIV Ag/AB combo assay, and the HIV-1 and HIV-2 RNA Viral Load COBAS 5800 platform. For positive or inconclusive results, the Geenius HIV1/2 confirmatory assay will be used. In all study visits, samples will be stored for additional retrospective HIV testing at the study central lab at INI-Fiocruz, as necessary. At any point of the study follow-up, participants with suspected HIV infection will be referred for follow-up investigation under study protocol. If HIV infection is confirmed at any point, CAB-LA injection will be stopped, and the participant will be immediately linked to care and ART initiation (according to the Brazilian guidelines) and will be invited to maintain a 1-year follow-up in the study with quarterly visits (step 4). Resistance testing will also be performed (if feasible, based on the number of viral copies).

**Table 2:** Overview of the ImPrEP CAB Brasil study visits and schedule of procedures: step 2 (year 2).

Procedures			Month 15		Month 17		Month 19		Month 21		Month 23		Month 25 or year 2
**Administrative, behavioral, and clinical**													
	Locator information	✓		✓		✓		✓		✓		✓	
	HIV counseling	✓		✓		✓		✓		✓		✓	
	Reproductive counseling	✓^a^		✓^a^		✓^a^		✓^a^		✓^a^		✓^a^	
	Condom, lubricant, and HIVST^b^	✓		✓		✓		✓		✓		✓	
	Adherence counseling	✓		✓		✓		✓		✓		✓	
	HIV Risk and Mental health assessment	✓		✓		✓		✓		✓		✓	
	Medical history	✓		✓		✓		✓		✓		✓	
	Concomitant medication	✓		✓		✓		✓		✓		✓	
	Directed physical examination	✓		✓		✓		✓		✓		✓	
	Weight and vital Signs	✓		✓		✓		✓		✓		✓	
	Height											✓	
	HIVST report	✓		✓		✓		✓		✓		✓	
**Laboratory**													
	Third-generation HIV rapid test	✓		✓		✓		✓		✓		✓	
	HIV RNA PCR^c^ (GeneXpert)											✓	
	HBV^d^ rapid test					✓						✓	
	HCV^e^ rapid test					✓						✓	
	Syphilis test^f^	✓				✓				✓		✓	
	Liver function test (ALT^g^)					✓						✓	
	Urine CT^h^ and NG^i^ test	✓				✓				✓		✓	
	Oropharyngeal CT and NG test	✓				✓				✓		✓	
	Rectal swab CT and NG test	✓				✓				✓		✓	
	Urine pregnancy test	✓^a^		✓^a^		✓^a^		✓^a^		✓^a^		✓^a^	
	Plasma and Serum storage	✓		✓		✓		✓		✓		✓	
**Study medication**													
	Injection	✓		✓		✓		✓		✓		✓	
	ISR^j^ evaluation	✓		✓		✓		✓		✓		✓	

^a^Only for transgender men.

^b^HIVST: HIV self-testing.

^c^PCR: polymerase chain reaction.

^d^HBV: hepatitis B.

^e^HCV: hepatitis C.

^f^Rapid test plus nontreponemal syphilis testing, as indicated.

^g^ALT: alanine aminotransferase.

^h^CT: *Chlamydia trachomatis*.

^i^NG: *Neisseria gonorrhea*.

^j^ISR: injection site reaction.

##### Randomized Injection Reminders via WhatsApp

As part of the consent process for enrollment in step 2, participants will be informed that they may be selected to receive a WhatsApp appointment reminder 2 days before each scheduled injection visit. Participants will be randomly assigned (1:1) to either the SOC (verbal communication and paper appointment slip letting participants know when they should come back to the clinic) or SOC plus WhatsApp arm using simple or block randomization and will be informed of which arm they are assigned to during the step 2 enrollment visit. During the month 5 visit, participants who were randomized to receive WhatsApp appointment reminders will be asked to complete a brief participant survey to assess the acceptability of receiving WhatsApp appointment reminders. If a participant does not return for their month 5 visit, the survey will be administered at their next clinic visit.

##### Switching From Oral TDF and FTC PrEP to CAB-LA PrEP

Step 1 participants may choose to switch from oral TDF and FTC PrEP to CAB-LA PrEP only once during the study period. These participants will undergo all step 2 procedures prior to receiving the first CAB-LA injection. Individuals with hepatitis B surface antigen–positive results and indication of hepatitis B treatment will be counseled to remain on oral TDF and FTC for PrEP. If hepatitis B treatment is not indicated and the individual chooses to switch to CAB-LA, enrollment will be allowed unless exclusionary hepatic criteria are identified (alanine aminotransferase ≥5 × upper limit of normal).

##### Specific Visit Procedures

Participants included in step 2 to receive CAB-LA PrEP who decide to discontinue participation before completing the minimum 1 year and maximum 2 years of study follow-up will complete early discontinuation visit procedures (Table 3). These participants will be invited to participate in a 12-month extension follow-up to safely monitor the CAB-LA “tail” phase and will be offered oral PrEP (TDF and FTC). Some of them will be invited to participate in a qualitative interview to describe their experience with CAB-LA PrEP and the reason for decision to discontinue CAB-LA PrEP.

If a participant misses an appointment but wishes to continue with CAB-LA PrEP, reloading with CAB-LA may be necessary, and the participant will need repeat HIV rapid tests before receiving a new CAB-LA injection. Oral bridging with TDF and FTC will be offered to participants who anticipate not being able to attend their scheduled injections (planned missed injections) in the +7 or −7-day window, starting approximately 2 months after the last injection dose of CAB-LA.

**Table 3:** The ImPrEP CAB Brasil–specific visit procedures.

Procedures			Final visit		Early discontinuation^a^		Seroconversion		STI^b^ interim visit		AE^c^ interim visit
**Administrative, behavioral, and clinical**											
	HIV counseling	✓		✓		✓		✓			
	Reproductive counseling^d^	✓		✓		✓^e^		✓^e^		✓^e^	
	Condom, lubricant, and HIVST^f^	✓		✓		✓		✓			
	Adherence counseling	✓		✓		✓					
	HIV Risk and Mental health assessment	✓		✓		✓				✓	
	Medical history	✓		✓		✓		✓		✓	
	Concomitant medication	✓		✓		✓		✓		✓	
	Physical examination	✓		✓		✓		✓		✓	
	Weight and vital Signs	✓		✓		✓					
	Height	✓		✓		✓					
	HIVST report	✓		✓^g^		✓^g^		✓^g^		✓^g^	
**Laboratory**											
	Third-generation HIV rapid test	✓		✓		✓		✓			
	HIV RNA PCR^h^ (GeneXpert)	✓		✓				✓^i^			
	HIV RNA PCR					✓					
	HBV^j^ rapid test	✓		✓		✓		✓			
	HCV^k^ rapid test	✓		✓		✓		✓			
	Syphilis test	✓		✓		✓		✓			
	Liver function test (ALT^l^)	✓		✓		✓				✓^m^	
	Creatinine or ClCr^n^	✓^o^		✓^o^							
	Oropharyngeal CT^p^ and NG^q^ test	✓		✓		✓		✓			
	Urine CT and NG test	✓		✓		✓		✓			
	Rectal swab CT and NG test	✓		✓		✓		✓			
	Urine pregnancy test	✓^r^		✓^r^		✓^r^		✓^e^		✓^e^	
	CD4 cell count					✓					
	HIV resistance testing					✓					
	Plasma and serum storage	✓		✓		✓					
**Study medication**											
	Injection	✓^d^									
	ISR^s^ evaluation	✓^d^								✓^t^	
	ART^u^ initiation					✓					
	TDF^v^ and FTC^w^ PrEP^x^	✓^y^		✓^y^							

^a^Any discontinuation occurring before the anticipated final study visit and not related to seroconversion.

^b^STI: sexually transmitted infection.

^c^AE: adverse event.

^d^Depending on the scenario that presents itself at the time of the final study visit.

^e^If pregnancy is suspected.

^f^HIVST: HIV self-test.

^g^If available.

^h^PCR: polymerase chain reaction.

^i^If HIV infection is suspected.

^j^HBV: hepatitis B.

^k^HCV: hepatitis C.

^l^ALT: alanine aminotransferase.

^m^Interim visits performed because of suspected hepatic adverse events should collect liver function tests.

^n^ClCr: creatinine clearance.

^o^If oral preexposure prophylaxis is initiated.

^p^CT: *Chlamydia trachomatis.*

^q^NG: *Neisseria gonorrhea.*

^r^Only for transgender men.

^s^ISR: injection site reaction.

^t^Interim visits performed because injection site reactions should follow specific protocol instructions.

^u^ART: antiretroviral therapy.

^v^TDF: tenofovir disoproxil fumarate.

^w^FTC: emtricitabine.

^x^PrEP: preexposure prophylaxis.

^y^In a final study visit or an early discontinuation visit, if the participant chooses to switch to oral preexposure prophylaxis, they will be followed in the 12-month follow-up study.

#### Study Step 3

Participants from step 2 will be invited to participate in step 3 if they meet the following eligibility requirements: (1) the participant decides to switch from CAB-LA to oral TDF and FTC during study follow-up or (2) the participant decides to early discontinue CAB-LA injections but does not want to start oral PrEP.

Participants who choose to switch from CAB-LA to oral TDF and FTC will need to complete the procedures related to the daily oral TDF and FTC enrollment visit (Table 3). The procedures are the same as those specified under both the early discontinuation visit and the final study visit, plus creatinine dosage and estimated creatinine clearance. Once enrolled in step 3, the follow-up consists of visits scheduled for the following intervals: month 1, month 4, month 7, month 10, and month 13 or year 1 (Table 4).

Participants who choose to keep off PrEP will be invited to be followed up in step 3. Participants will have quarterly visits with sexual risk assessment, HIV and STI testing and counseling, and the provision of condoms and lubricants.

**Table 4:** The ImPrEP CAB Brasil step 3 procedures: daily oral preexposure prophylaxis.

Procedures			Enrollment^a^		Month 1		Month 4		Month 7		Month 10		Month 13 or year 1
**Administrative, behavioral, and clinical**													
	Locator information	✓		✓		✓		✓		✓		✓	
	HIV risk assessment and counseling	✓		✓		✓		✓		✓		✓	
	Condom, lubricant, and HIVST^b^	✓		✓		✓		✓		✓		✓	
	Adherence counseling	✓		✓		✓		✓		✓		✓	
	Medical history	✓		✓		✓		✓		✓		✓	
	Concomitant medication	✓		✓		✓		✓		✓		✓	
	Directed physical examination	✓		✓		✓		✓		✓		✓	
	Weight and vital Signs	✓		✓		✓		✓		✓		✓	
	Height	✓										✓	
	HIVST report			✓		✓		✓		✓		✓	
**Laboratory**													
	Third-generation HIV rapid test	✓		✓		✓		✓		✓		✓	
	HBV^c^ rapid test	✓						✓				✓	
	HCV^d^ rapid test	✓						✓				✓	
	Syphilis test	✓				✓		✓		✓		✓	
	Liver function test (ALT^e^)^f^	✓						✓				✓	
	Creatinine or ClCr^g^	✓						✓				✓	
	Oropharyngeal CT^h^ and NG^i^ test	✓				✓		✓				✓	
	Urine CT and NG test	✓				✓		✓		✓			
	Rectal swab CT and NG	✓				✓		✓		✓			
	Plasma and serum storage	✓		✓		✓		✓		✓		✓	
**PrEP^j^ medication**													
	TDF^k^ and FTC^l^ dispensation	✓		✓		✓		✓		✓		✓	

^a^Enrollment procedures for oral preexposure prophylaxis are the same as those of the final study visit and early discontinuation visit.

^b^HIVST: HIV self-test.

^c^HBV: hepatitis B.

^d^HCV: hepatitis C.

^e^ALT: alanine aminotransferase.

^f^Only for those who switched from long-acting injectable cabotegravir.

^g^ClCr: creatinine clearance.

^h^CT: *Chlamydia trachomatis.*

^i^NG: *Neisseria gonorrhea*.

^j^PrEP: preexposure prophylaxis.

^k^TDF: tenofovir disoproxil fumarate.

^l^FTC: emtricitabine.

#### Step 4

##### Overview

Participants who are diagnosed with HIV while using CAB-LA during study follow-up will perform the procedures related to the *seroconversion visit* (Table 3) and will be invited to join step 4. HIV treatment will be initiated in accordance with the Brazilian guidelines. The participant will be requested to come to the study clinic for quarterly visits for 1 year. HIV RNA polymerase chain reaction will be evaluated in all visits. If a participant is identified with suspected virological failure, a new resistance test will be performed, and a new ART regimen will be selected based on the resistance test results.

##### Qualitative Assessments During the Study

At month 7, a select set of participants will be invited to participate in a semistructured qualitative interview guided by the TFA to understand their experiences with CAB-LA PrEP and with the clinic services, including WhatsApp appointment reminders and HIV self-testing. Up to 16 participants will be interviewed at each site. The aim is to sample approximately 8 individuals who continually adhered to their injection schedule, approximately 4 individuals who needed to reload, and 4 individuals who discontinued CAB-LA injections. Interviews will be conducted in Portuguese and audio recorded. Interviewers will complete a debriefing report summarizing the key points of the interview within 12 hours of completing the interview. Audio recordings will be transcribed *verbatim* by study staff.

##### Health Care Provider Survey

At the end of the implementation study, health providers will be asked to complete an in-depth interview on their experiences integrating CAB-LA in the existing PrEP services and their views on the mHealth tool and WhatsApp reminders. The interview questionnaire will seek to understand the facilitators of and barriers to CAB-LA service delivery and will be informed by implementation science constructs drawn from the TFA (affective attitude, burden, ethicality, intervention coherence, opportunity cost or relative advantage, perceived effectiveness, and self-efficacy) [29], Reach, Effectiveness, Adoption, Implementation, and Maintenance (implementation) [30], and Proctor et al [31] (feasibility). The definitions of the constructs are summarized in Multimedia Appendix 1. During these interviews, health care providers will also be asked to review their most recent process mapping, describe changes that happened with CAB-LA implementation, and describe ongoing “pain points." Short Likert-scale surveys will also be embedded in the questionnaires to gather quantitative data on acceptability.

### Major Outcomes

The major clinical and implementation outcomes, levels of analysis, methods, and timing are described in Table 5.

**Table 5:** Summary of major clinical and implementation outcomes.

Major outcomes		Level	Methods and timing
**Clinical**			
	HIV incidence (effectiveness)	Individual	HIV test completed at each visit and interim analysis after 12 months of enrollment and upon the completion of the study
	Resistance to CAB-LA^a^ or oral TDF^b^ and FTC^c^ PrEP^d^ (effectiveness and safety)	Individual	HIV genotyping test at the time of seroconversion or suspected viral failure at any time during the 12-month follow-up
**Implementation**			
	mHealth^e^ intervention acceptability (from TFA^f^)	Individual participant (client or potential PrEP user)	Survey of all participants receiving mHealth intervention immediately upon the completion of SOC^g^ or SOC and mHealth education and counseling Qualitative interview (12 participants per site who receive mHealth and SOC) during the enrollment period for step 1
	mHealth intervention effectiveness (from RE-AIM^h^)	Individual participant (client or potential PrEP user)	Knowledge survey of all participants receiving mHealth intervention immediately upon the completion of SOC or SOC and mHealth education and counseling Qualitative interviews of 8 participants per site who receive SOC counseling and 12 participants who receive SOC and mHealth education and counseling about their knowledge and beliefs about oral and CAB-LA PrEP in relation to their choice Adherence data at the end of the study
	mHealth tool acceptability (TFA) and feasibility (from Proctor et al [31])	Individual provider and clinic setting	Interviews at the end of the study including questions that will generate both qualitative and quantitative data
	WhatsApp appointment reminder acceptability (TFA)	Individual client or PrEP user	Brief survey given to each participant who received WhatsApp appointment reminders at their month 5 visit Qualitative interviews (16 participants per site) conducted at month 7
	WhatsApp appointment reminder effectiveness (from RE-AIM)	Individual client or PrEP user	Study adherence data at the middle and end of the study Qualitative interviews (16 participants per site) conducted at month 7
	WhatsApp appointment reminder feasibility (Proctor et al [31]) and implementation (RE-AIM)	Individual provider and clinic setting	Interviews at the end of the study including questions that will generate both qualitative and quantitative data WhatsApp appointment reminder program data files
	Acceptability (TFA) of STI^i^ self-test	Individual client or CAB-LA PrEP user	Qualitative interviews (16 participants per site) conducted at month 7
	Acceptability (TFA) and feasibility (Proctor) of STI self-test	Individual provider and clinic setting	Qualitative interviews at the end of the study
	Acceptability (TFA) of HIV self-testing	Individual client or CAB- LA PrEP user	Qualitative interviews (16 participants per site) conducted at month 7
	Implementation: barriers to and facilitators of the integrated delivery of CAB-LA PrEP in the existing oral PrEP services	Individual participant (client or CAB-LA PrEP user) Individual provider and clinic setting	Qualitative interviews (16 participants per site) conducted at month 7 Interviews at the end of the study including questions that will generate both qualitative and quantitative data
	Feasibility of CAB-LA PrEP in public clinic PrEP services	Individual provider and clinic setting	Interviews at the end of the study including questions that will generate both qualitative and quantitative data Review of routine study documentation
	Maintenance	Individual participant (client or CAB-LA PrEP user)	Adherence to injection visits at the middle and end of the study
	Reach	Individual participant and cohort characteristics	Survey of all participants receiving mHealth intervention immediately upon the completion of SOC or SOC and mHealth education and counseling about the rationale for their PrEP choice Qualitative interview (12 participants per site who receive mHealth and SOC) during the enrollment period for step 1 that will include questions about the rational for their PrEP choice Study data on the number of participants enrolled and pace of enrollment and demographic data

^a^CAB-LA: long-acting injectable cabotegravir.

^b^TDF: tenofovir disoproxil fumarate.

^c^FTC: emtricitabine.

^d^PrEP: preexposure prophylaxis.

^e^mHealth: mobile health.

^f^TFA: theoretical framework for acceptability.

^g^SOC: standard of care.

^h^RE-AIM: Reach, Effectiveness, Adoption, Implementation, and Maintenance.

^i^STI: sexually transmitted infection.

### Statistical Analysis

Our major clinical outcome (primary analysis) will adopt an intent-to-treat approach and compare HIV incidence (and 95% CI) among individuals in the CAB-LA cohort with HIV incidence (and 95% CI) among individuals on daily PrEP within the Brazilian SUS. We will estimate the rate of HIV incidence with 95% CI in each group using Kaplan-Maier statistics. We will use a Cox proportional hazard model to assess the relative hazard (hazard ratio with 95% CI) between the 2 groups, overall and by site, age category, and gender.

It is anticipated that there will be an average follow-up of 1.5 years per person with at least 75% retention each year (N=1350 person-years of follow-up) and that incidence will be higher than that observed in HPTN 083 in Latin America (0.60 per 100 person-years). If the observed incidence is approximately 50% higher than that observed in HPTN 083, the study will be able to estimate an incidence of 0.85 (95% CI of 0.47-1.42) per 100 person-years (Table 6). If the observed incidence is approximately twice as high as that observed in HPTN 083, then the study will be able to estimate an incidence of 1.18 (95% CI of 0.72-1.82) per 100 person-years. If the comparison group is of similar size, then the incidence would need to be 2.2 to 2.5 times higher to observe statistically significant differences between CAB-LA and oral PrEP. However, if the comparison group is 3 times larger, then the incidence would need to be 2 to 2.2 times higher to observe statistically significant differences between CAB-LA and oral PrEP.

To reach the primary implementation objective, we will report descriptive statistics (numbers, proportions, and medians) of Likert-scale responses to the TFA constructs (affective attitude, burden, ethicality, intervention coherence, opportunity costs, and perceived effectiveness and self-efficacy) among health care providers.

**Table 6:** Observable differences given potential HIV incidence in intervention and comparison groups.

Group				Incidence (95% CI)
**Intervention: CAB-LA^a^ **PrEP**^b^**				
	**1350 py^c^ incidence/100 py**			
		8 cases	0.59 (0.28-1.13)	
		12 cases	0.89 (0.48-1.51)	
		18 cases	1.19 (0.71-1.88)	
**Comparison: oral PrEP in the Brazilian health system**				
	**1350 py^c^ incidence/100 py**			
		24 cases	1.78 (1.17-2.61)	
		30 cases	2.22 (1.53-3.13)	
		36 cases	2.67 (1.90-3.65)	
	**2700 py incidence/100 py**			
		43 cases	1.59 (1.17-2.13)	
		54 cases	2.00 (1.52-2.59)	
		66 cases	2.44 (1.91-3.09)	
	**4050 py incidence/100 py**			
		60 cases	1.48 (1.14-1.89)	
		78 cases	1.93 (1.53-2.39)	
		94 cases	2.32 (1.89-2.83)	

^a^CAB-LA: long-acting cabotegravir.

^b^PrEP: preexposure prophylaxis.

^c^py: person-years.

### Ethics Approval

The study protocol and informed consent forms were reviewed and approved by the Evandro Chagas National Institute of Infectious Diseases (INI), Fundação Oswaldo Cruz (Fiocruz) institutional review board (August 25, 2022; #CAAE 59166522.7.1001.5262) and the Research Ethics Review Committee at the WHO with respect to scientific content and compliance with applicable research and human participants’ regulations. The protocol, site-specific informed consent forms, participant education and recruitment materials, and any requested documents and their subsequent modifications were also be reviewed and approved by the ethical review bodies responsible for the oversight of the research conducted at each of the study sites. Informed consent will be obtained in 2 study steps. In step 1, consent will be obtained from participants who visit the site looking for PrEP. In step 2, the participants will provide consent for the use of injectable CAB-LA PrEP. This study may be paused or stopped at any time by the local institutional review board or the study’s principal investigator.

## Results

During the third and fourth quarters of 2022, we obtained regulatory approvals; programmed data entry or management systems; trained sites; and performed community consultancy, process mapping, and formative work. Study enrollment is anticipated to happen in the first quarter of 2023. The planned activities are presented in Table 7.

**Table 7:** Planned activities for the ImPrEP CAB Brasil study (2022-2024).

Activities	Quarter 2 of 2022	Quarter 3 of 2022	Quarter 4 of 2022	Quarter 1 of 2023	Quarter 2 of 2023	Quarter 3 of 2023	Quarter 4 of 2023	Quarter 1 of 2024	Quarter 2 of 2024	Quarter 3 of 2024	Quarter 4 of 2024
Regulatory submission	✓										
Regulatory approvals		✓	✓								
Data entry or management systems programmed	✓	✓									
Drug importation and distribution				✓	✓						
Site equipping	✓	✓	✓								
SOP^a^ and SSP^b^ development	✓	✓	✓	✓							
Staff selection	✓	✓									
Staff training		✓	✓								
Process mapping			✓								
Formative work		✓	✓	✓							
Enrollment					✓	✓	✓				
Follow-up						✓	✓	✓	✓	✓	
Data consolidation							✓	✓	✓	✓	
Data analysis										✓	✓
Final report											✓
Manuscript submission											✓

^a^SOP: standard operational procedures.

^b^SSP: study-specific plan.

## Discussion

### Principal Findings

ImPrEP CAB Brasil is the first study to evaluate CAB-LA PrEP implementation in Latin America. Latin America is one of the regions where PrEP scale-up, as well as the reduction of inequalities in PrEP access, is most needed. We hereby describe the study protocol, providing details of the study’s objectives, procedures, outcomes, and statistical analysis to aid researchers from Brazil, Latin America, and other regions during CAB-LA implementation within public health systems.

To date, no clinical or implementation results are available for injectable PrEP with CAB-LA in real-life settings. We expect the acceptability of CAB-LA in Brazil to be high. Data from a web-based survey and descriptive choice experiment have shown that injectable PrEP was preferred over other PrEP modalities in Brazil [35-37]. Our results regarding PrEP choice among individuals seeking PrEP in health units will increase this body of evidence. For instance, in the field of contraception, the increased availability of options and informed decision-making in the choice of contraception have improved the uptake and persistence of the methods used [38]. It is likely that expanding the method options for PrEP will similarly improve uptake and persistence.

Our study will also contribute to the expansion of the knowledge on the best HIV testing algorithm to be implemented in low- and middle-income countries when using CAB-LA for PrEP, which is a major need.

This study is coordinated by INI-Fiocruz, an internationally recognized research institution and the leading reference center in Latin America for infectious diseases. INI-Fiocruz has been working closely with the Brazilian Ministry of Health (BMOH), the Pan American Health Organization, Unitaid, and the WHO to address evidence gaps and identify key learnings to inform the introduction of injectable CAB-LA PrEP among the most susceptible populations. The INI-Fiocruz team has extensive experience in engaging SGMs in demand creation, providing HIV combination prevention services that foster PrEP uptake and retention and advocating for enhanced HIV prevention policies [39,40]. In addition to being a recognized academic institution, INI-Fiocruz is the largest HIV prevention and treatment center in Rio de Janeiro, Brazil, with over 1000 SGMs tested per year [41]. The INI-Fiocruz study team was responsible for planning, designing, and implementing the PrEP Brasil study [14], which provided critical evidence to support the BMOH in the incorporation of PrEP as a public health policy. The team also coordinated the ImPrEP study, which provided information for PrEP surveillance in Brazil, and PrEP implementation in Mexico and Peru [16].

This study is poised for informing policy makers, building public health staff capacity, and creating environments for successfully implementing the proposed prevention strategies in collaboration with the BMOH. Moreover, we expect the mHealth component to be highly acceptable and possibly implemented within the Brazilian public health system.

### Conclusions

The ImPrEP CAB Brasil study will contribute to the generation of valuable information to inform the design of programmatic strategies for implementing CAB-LA PrEP in Brazil, maximizing the public health impact of PrEP in reducing HIV incidence among young SGMs in Brazil, Latin America, and other countries in the Global South.

## Appendix

Multimedia Appendix 1 Additional information about ImPrEP CAB-Brasil protocol.

## Abbreviations

ART: antiretroviral therapy
BMOH: Brazilian Ministry of Health
CAB-LA: long-acting injectable cabotegravir
FGD: focus group discussion
Fiocruz: Fundação Oswaldo Cruz
FTC: emtricitabine
HPTN: HIV Prevention Trials Network
INI: Instituto Nacional de Infectologia Evandro Chagas
mHealth: mobile health
MSM: gay men, bisexual men, and other men who have sex with men
PrEP: preexposure prophylaxis
SGM: sexual and gender minority
SOC: standard of care
STI: sexually transmitted infection
SUS: Sistema Único de Saúde
TDF: tenofovir disoproxil fumarate
TFA: theoretical framework for acceptability
TGW: travesti and transgender women
WHO: World Health Organization

## Data Availability

This manuscript is a description of a study protocol; therefore, no data are available.

## References

[1] (2021). UNAIDS Data 2021. Joint United Nations Programme on HIV/AIDS.

[2] Coelho LE, Torres TS, Veloso VG, Grinsztejn B, Jalil EM, Wilson EC, McFarland W (2021). The prevalence of HIV among men who have sex with men (MSM) and young MSM in Latin America and the Caribbean: a systematic review. AIDS Behav.

[3] Bastos FI, Bastos LS, Coutinho C, Toledo L, Mota JC, Velasco-de-Castro CA, Sperandei S, Brignol S, Travassos TS, Dos Santos CM, Malta MS, “Divas Research Group” (2018). HIV, HCV, HBV, and syphilis among transgender women from Brazil: assessing different methods to adjust infection rates of a hard-to-reach, sparse population. Medicine (Baltimore).

[4] Grinsztejn B, Jalil EM, Monteiro L, Velasque L, Moreira RI, Garcia AC, Castro CV, Krüger A, Luz PM, Liu AY, McFarland W, Buchbinder S, Veloso VG, Wilson EC, Transcender Study Team (2017). Unveiling of HIV dynamics among transgender women: a respondent-driven sampling study in Rio de Janeiro, Brazil. Lancet HIV.

[5] Luz PM, Benzaken A, de Alencar TM, Pimenta C, Veloso VG, Grinsztejn B (2018). PrEP adopted by the Brazilian national health system: what is the size of the demand?. Medicine (Baltimore).

[6] (2022). Painel PrEP. Ministério da Saúde Brasil.

[7] Sullivan A, Chiavenna C, Cartier A, Jaafar S, Mitchell H, Ogaz D, Mason E, Osman R, Coukan F, Diamente V, Golombek R (2021). BPD2/7 HIV and STI incidence among MSM users and non-users of HIV PrEP in England: results from the Impact trial. Proceedings of the 18th European AIDS Conference.

[8] Scheer S, Hsu L, Schwarcz S, Pipkin S, Havlir D, Buchbinder S, Hessol NA (2018). Trends in the San Francisco human immunodeficiency virus epidemic in the "getting to zero" era. Clin Infect Dis.

[9] Grulich AE, Jin F, Bavinton BR, Yeung B, Hammoud MA, Amin J, Cabrera G, Clackett S, Ogilvie E, Vaccher S, Vickers T, McNulty A, Smith DJ, Dharan NJ, Selvey C, Power C, Price K, Zablotska I, Baker DA, Bloch M, Brown K, Carmody CJ, Carr A, Chanisheff D, Doong N, Finlayson R, Lewis DA, Lusk J, Martin S, Ooi C, Read P, Ryder N, Smith D, Tuck Meng Soo C, Templeton DJ, Vlahakis E, Guy R, Expanded PrEP Implementation in Communities New South Wales (EPIC-NSW) research group (2021). Long-term protection from HIV infection with oral HIV pre-exposure prophylaxis in gay and bisexual men: findings from the expanded and extended EPIC-NSW prospective implementation study. Lancet HIV.

[10] Grulich AE, Guy R, Amin J, Jin F, Selvey C, Holden J, Schmidt HM, Zablotska I, Price K, Whittaker B, Chant K, Cooper C, McGill S, Telfer B, Yeung B, Levitt G, Ogilvie EE, Dharan NJ, Hammoud MA, Vaccher S, Watchirs-Smith L, McNulty A, Smith DJ, Allen DM, Baker D, Bloch M, Bopage RI, Brown K, Carr A, Carmody CJ, Collins KL, Finlayson R, Foster R, Jackson EY, Lewis DA, Lusk J, O'Connor CC, Ryder N, Vlahakis E, Read P, Cooper DA, Expanded PrEP Implementation in Communities New South Wales (EPIC-NSW) research group (2018). Population-level effectiveness of rapid, targeted, high-coverage roll-out of HIV pre-exposure prophylaxis in men who have sex with men: the EPIC-NSW prospective cohort study. Lancet HIV.

[11] Koss CA, Havlir DV, Ayieko J, Kwarisiima D, Kabami J, Chamie G, Atukunda M, Mwinike Y, Mwangwa F, Owaraganise A, Peng J, Olilo W, Snyman K, Awuonda B, Clark TD, Black D, Nugent J, Brown LB, Marquez C, Okochi H, Zhang K, Camlin CS, Jain V, Gandhi M, Cohen CR, Bukusi EA, Charlebois ED, Petersen ML, Kamya MR, Balzer LB (2021). HIV incidence after pre-exposure prophylaxis initiation among women and men at elevated HIV risk: a population-based study in rural Kenya and Uganda. PLoS Med.

[12] Haberer JE, Bangsberg DR, Baeten JM, Curran K, Koechlin F, Amico KR, Anderson P, Mugo N, Venter F, Goicochea P, Caceres C, O'Reilly K (2015). Defining success with HIV pre-exposure prophylaxis: a prevention-effective adherence paradigm. AIDS.

[13] Laborde ND, Kinley PM, Spinelli M, Vittinghoff E, Whitacre R, Scott HM, Buchbinder SP (2020). Understanding PrEP persistence: provider and patient perspectives. AIDS Behav.

[14] Grinsztejn B, Hoagland B, Moreira RI, Kallas EG, Madruga JV, Goulart S, Leite IC, Freitas L, Martins LM, Torres TS, Vasconcelos R, De Boni RB, Anderson PL, Liu A, Luz PM, Veloso VG, PrEP Brasil Study Team (2018). Retention, engagement, and adherence to pre-exposure prophylaxis for men who have sex with men and transgender women in PrEP Brasil: 48 week results of a demonstration study. Lancet HIV.

[15] Konda KA, Torres TS, Mariño G, Ramos A, Moreira RI, Leite IC, Cunha M, Jalil EM, Hoagland B, Guanira JV, Benedetti M, Pimenta C, Vermandere H, Bautista-Arredondo S, Vega-Ramirez H, Veloso VG, Caceres CF, Grinsztejn B, ImPrEP Study Group (2022). Factors associated with long-term HIV pre-exposure prophylaxis engagement and adherence among transgender women in Brazil, Mexico and Peru: results from the ImPrEP study. J Int AIDS Soc.

[16] Veloso VG, Cáceres CF, Hoagland B, Moreira RI, Vega-Ramírez H, Konda KA, Leite IC, Bautista-Arredondo S, Vinícius de Lacerda M, Valdez Madruga J, Farias A, Lima JN, Zonta R, Lauria L, Tamayo CV, Flores HJ, Santa Cruz YM, Aguayo RM, Cunha M, Moreira J, Makkeda AR, Díaz S, Guanira JV, Vermandere H, Benedetti M, Ingold HL, Pimenta MC, Torres TS, Grinsztejn B, ImPrEP Study Group (2023). Same-day initiation of oral pre-exposure prophylaxis among gay, bisexual, and other cisgender men who have sex with men and transgender women in Brazil, Mexico, and Peru (ImPrEP): a prospective, single-arm, open-label, multicentre implementation study. Lancet HIV.

[17] Serota DP, Rosenberg ES, Lockard AM, Rolle CP, Luisi N, Cutro S, Del Rio C, Siegler AJ, Sanchez TH, Sullivan PS, Kelley CF (2018). Beyond the biomedical: preexposure prophylaxis failures in a cohort of young black men who have sex with men in Atlanta, Georgia. Clin Infect Dis.

[18] Scott HM, Spinelli M, Vittinghoff E, Morehead-Gee A, Hirozawa A, James C, Hammer H, Liu A, Gandhi M, Buchbinder S (2019). Racial/ethnic and HIV risk category disparities in preexposure prophylaxis discontinuation among patients in publicly funded primary care clinics. AIDS.

[19] Deutsch MB, Glidden DV, Sevelius J, Keatley J, McMahan V, Guanira J, Kallas EG, Chariyalertsak S, Grant RM, iPrEx investigators (2015). HIV pre-exposure prophylaxis in transgender women: a subgroup analysis of the iPrEx trial. Lancet HIV.

[20] Celum C, Hosek S, Tsholwana M, Kassim S, Mukaka S, Dye BJ, Pathak S, Mgodi N, Bekker LG, Donnell DJ, Wilson E, Yuha K, Anderson PL, Agyei Y, Noble H, Rose SM, Baeten JM, Fogel JM, Adeyeye A, Wiesner L, Rooney J, Delany-Moretlwe S (2021). PrEP uptake, persistence, adherence, and effect of retrospective drug level feedback on PrEP adherence among young women in southern Africa: results from HPTN 082, a randomized controlled trial. PLoS Med.

[21] Spinelli MA, Laborde N, Kinley P, Whitacre R, Scott HM, Walker N, Liu AY, Gandhi M, Buchbinder SP (2020). Missed opportunities to prevent HIV infections among pre-exposure prophylaxis users: a population-based mixed methods study, San Francisco, United States. J Int AIDS Soc.

[22] Jin F, Amin J, Guy R, Vaccher S, Selvey C, Zablotska I, Holden J, Price K, Yeung B, Ogilvie E, Quichua GC, Clackett S, McNulty A, Smith D, Templeton DJ, Bavinton B, Grulich AE, Expanded PrEP Implementation in Communities New South Wales (EPIC-NSW) research group (2021). Adherence to daily HIV pre-exposure prophylaxis in a large-scale implementation study in New South Wales, Australia. AIDS.

[23] Krakower D, Maloney KM, Powell VE, Levine K, Grasso C, Melbourne K, Marcus JL, Mayer KH (2019). Patterns and clinical consequences of discontinuing HIV preexposure prophylaxis during primary care. J Int AIDS Soc.

[24] Coelho LE, Torres TS, Veloso VG, Landovitz RJ, Grinsztejn B (2019). Pre-exposure prophylaxis 2.0: new drugs and technologies in the pipeline. Lancet HIV.

[25] Delany-Moretlwe S, Hughes JP, Bock P, Ouma SG, Hunidzarira P, Kalonji D, Kayange N, Makhema J, Mandima P, Mathew C, Spooner E, Mpendo J, Mukwekwerere P, Mgodi N, Ntege PN, Nair G, Nakabiito C, Nuwagaba-Biribonwoha H, Panchia R, Singh N, Siziba B, Farrior J, Rose S, Anderson PL, Eshleman SH, Marzinke MA, Hendrix CW, Beigel-Orme S, Hosek S, Tolley E, Sista N, Adeyeye A, Rooney JF, Rinehart A, Spreen WR, Smith K, Hanscom B, Cohen MS, Hosseinipour MC, HPTN 084 study group (2022). Cabotegravir for the prevention of HIV-1 in women: results from HPTN 084, a phase 3, randomised clinical trial. Lancet.

[26] Landovitz RJ, Donnell D, Clement ME, Hanscom B, Cottle L, Coelho L, Cabello R, Chariyalertsak S, Dunne EF, Frank I, Gallardo-Cartagena JA, Gaur AH, Gonzales P, Tran HV, Hinojosa JC, Kallas EG, Kelley CF, Losso MH, Madruga JV, Middelkoop K, Phanuphak N, Santos B, Sued O, Valencia Huamaní J, Overton ET, Swaminathan S, Del Rio C, Gulick RM, Richardson P, Sullivan P, Piwowar-Manning E, Marzinke M, Hendrix C, Li M, Wang Z, Marrazzo J, Daar E, Asmelash A, Brown TT, Anderson P, Eshleman SH, Bryan M, Blanchette C, Lucas J, Psaros C, Safren S, Sugarman J, Scott H, Eron JJ, Fields SD, Sista ND, Gomez-Feliciano K, Jennings A, Kofron RM, Holtz TH, Shin K, Rooney JF, Smith KY, Spreen W, Margolis D, Rinehart A, Adeyeye A, Cohen MS, McCauley M, Grinsztejn B, HPTN 083 Study Team (2021). Cabotegravir for HIV prevention in cisgender men and transgender women. N Engl J Med.

[27] (2022). Guidelines on long-acting injectable cabotegravir for HIV prevention. World Health Organization.

[28] Landes SJ, McBain SA, Curran GM (2019). An introduction to effectiveness-implementation hybrid designs. Psychiatry Res.

[29] Sekhon M, Cartwright M, Francis JJ (2017). Acceptability of healthcare interventions: an overview of reviews and development of a theoretical framework. BMC Health Serv Res.

[30] Glasgow RE, Harden SM, Gaglio B, Rabin B, Smith ML, Porter GC, Ory MG, Estabrooks PA (2019). RE-AIM planning and evaluation framework: adapting to new science and practice with a 20-year review. Front Public Health.

[31] Proctor E, Silmere H, Raghavan R, Hovmand P, Aarons G, Bunger A, Griffey R, Hensley M (2011). Outcomes for implementation research: conceptual distinctions, measurement challenges, and research agenda. Adm Policy Ment Health.

[32] Wooldridge AR, Carayon P, Hoonakker P, Hose BZ, Eithun B, Brazelton 3rd T, Ross J, Kohler JE, Kelly MM, Dean SM, Rusy D, Gurses AP (2020). Work system barriers and facilitators in inpatient care transitions of pediatric trauma patients. Appl Ergon.

[33] Antonacci G, Reed JE, Lennox L, Barlow J (2018). The use of process mapping in healthcare quality improvement projects. Health Serv Manage Res.

[34] Trebble TM, Hansi N, Hydes T, Smith MA, Baker M (2010). Process mapping the patient journey: an introduction. BMJ.

[35] de Aguiar Pereira CC, Torres TS, Luz PM, Hoagland B, Farias A, Brito JD, Guimarães Lacerda MV, da Silva DA, Benedetti M, Pimenta MC, Grinsztejn B, Veloso VG (2021). Preferences for pre-exposure prophylaxis (PrEP) among men who have sex with men and transgender women at risk of HIV infection: a multicentre protocol for a discrete choice experiment in Brazil. BMJ Open.

[36] de Aguiar Pereira CC, Torres TS, Luz PM, Hoagland B, Farias A, Brito JD, Lacerda MV, Silva DA, Benedetti M, Pimenta MC, Grinsztejn B, Veloso VG (2023). Preferences for pre-exposure prophylaxis (PrEP) among sexual and gender minorities: a discrete choice experiment in Brazil. Lancet Reg Health Am.

[37] Torres TS, Nascimento AR, Coelho LE, Konda KA, Vega-Ramirez EH, Elorreaga OA, Diaz-Sosa D, Hoagland B, Guanira JV, Pimenta C, Benedetti M, Caceres CF, Veloso VG, Grinsztejn B (2023). Preferences for PrEP modalities among gay, bisexual, and other men who have sex with men from Brazil, Mexico, and Peru: a cross-sectional study. Ther Adv Infect Dis.

[38] Gray AL, Smit JA, Manzini N, Beksinska M (2006). Systematic review of contraceptive medicines “Does choice make a difference?”. Reproductive Health and HIV Research Unit.

[39] Bezerra DR, Jalil CM, Jalil EM, Coelho LE, Carvalheira E, Freitas J, Monteiro L, Santos T, Souza C, Hoagland B, Veloso VG, Grinsztejn B, Cardoso SW, Torres TS (2022). Complementary recruitment strategies to reach men who have sex with men and transgender women: the experience of a large Brazilian HIV prevention service. AIDS Behav.

[40] Bezerra DR, Jalil CM, Jalil EM, Coelho LE, Netto EC, Freitas J, Monteiro L, Santos T, Souza C, Hoagland B, Veloso VG, Grinsztejn B, Cardoso SW, Torres TS (2022). Comparing web-based venues to recruit gay, bisexual, and other cisgender men who have sex with men to a large HIV prevention service in Brazil: evaluation study. JMIR Form Res.

[41] Teixeira SL, Jalil CM, Jalil EM, Nazer SC, da Costs Cruz Silva S, Veloso VG, Luz PM, Grinsztejn B (2021). Evidence of an untamed HIV epidemic among MSM and TGW in Rio de Janeiro, Brazil: a 2018 to 2020 cross-sectional study using recent infection testing. J Int AIDS Soc.

