# Supplementary material for: Long-Acting Injectable Cabotegravir for HIV Preexposure Prophylaxis Among Sexual and Gender Minorities: Protocol for an Implementation Study
Source: JMIR Public Health Surveill. 2023 Apr 19;9:e44961. doi: 10.2196/44961 (PMC10157454; doi:10.2196/44961)
Supplement: Multimedia Appendix 1 [file publichealth_v9i1e44961_app1.docx]

**Supplementary Material**

1. Protocol Team Roster
2. Selected Implementation Science Constructs
3. Exclusion Criteria
4. Objectives and Endpoints
5. Interim Analysis
6. **Protocol Team Roster**

**Coordinating Site**

| **Name** | **Roles and Responsibilities** | **Contact Details** |
| --- | --- | --- |
| Beatriz Grinsztejn, MD, PhD | Study PI | **Institution:** Instituto Nacional de Infectologia Evandro Chagas - Fundação Oswaldo Cruz  [Avenida Brasil, 4365](https://maps.google.com/?q=Avenida+Brasil,+4365%0D+%0D+Manguinhos%0D+%0D+Rio+de+Janeiro,+Brasil+21040-360&entry=gmail&source=g), [Manguinhos](https://maps.google.com/?q=Avenida+Brasil,+4365%0D+%0D+Manguinhos%0D+%0D+Rio+de+Janeiro,+Brasil+21040-360&entry=gmail&source=g)  [Rio de Janeiro, Brasil 21040-360](https://maps.google.com/?q=Avenida+Brasil,+4365%0D+%0D+Manguinhos%0D+%0D+Rio+de+Janeiro,+Brasil+21040-360&entry=gmail&source=g)  Phone: 55-21-2270-7064  E-mail: [gbeatriz@ini.fiocruz.br](mailto:gbeatriz@ini.fiocruz.br) |
| Valdiléa Veloso, MD, PhD | Study Co-PI | **Institution**: Instituto Nacional de Infectologia Evandro Chagas - Fundação Oswaldo Cruz  [Avenida Brasil, 4365](https://maps.google.com/?q=Avenida+Brasil,+4365%0D+%0D+Manguinhos%0D+%0D+Rio+de+Janeiro,+Brasil+21040-360&entry=gmail&source=g), [Manguinhos](https://maps.google.com/?q=Avenida+Brasil,+4365%0D+%0D+Manguinhos%0D+%0D+Rio+de+Janeiro,+Brasil+21040-360&entry=gmail&source=g)  [Rio de Janeiro, Brasil 21040-360](https://maps.google.com/?q=Avenida+Brasil,+4365%0D+%0D+Manguinhos%0D+%0D+Rio+de+Janeiro,+Brasil+21040-360&entry=gmail&source=g)  Phone: 55-21-2270-7064  E-mail:  [valdilea.veloso@ini.fiocruz.br](mailto:valdilea.veloso@ini.fiocruz.br) |
| Brenda Hoagland, MD, PhD | Medical Officer | **Institution:** Instituto Nacional de Infectologia Evandro Chagas - Fundação Oswaldo Cruz  [Avenida Brasil, 4365](https://maps.google.com/?q=Avenida+Brasil,+4365%0D+%0D+Manguinhos%0D+%0D+Rio+de+Janeiro,+Brasil+21040-360&entry=gmail&source=g), [Manguinhos](https://maps.google.com/?q=Avenida+Brasil,+4365%0D+%0D+Manguinhos%0D+%0D+Rio+de+Janeiro,+Brasil+21040-360&entry=gmail&source=g)  [Rio de Janeiro, Brasil 21040-360](https://maps.google.com/?q=Avenida+Brasil,+4365%0D+%0D+Manguinhos%0D+%0D+Rio+de+Janeiro,+Brasil+21040-360&entry=gmail&source=g)  Phone: 55-21-38659122  E-mail: [brenda.hoagland@ini.fiocruz.br](mailto:brenda.hoagland@ini.fiocruz.br) |
| Emilia Jalil, MD, PhD | Co-investigator and technical advisor for transgender and non-binary research | **Institution:** Instituto Nacional de Infectologia Evandro Chagas - Fundação Oswaldo Cruz  [Avenida Brasil, 4365](https://maps.google.com/?q=Avenida+Brasil,+4365%0D+%0D+Manguinhos%0D+%0D+Rio+de+Janeiro,+Brasil+21040-360&entry=gmail&source=g), [Manguinhos](https://maps.google.com/?q=Avenida+Brasil,+4365%0D+%0D+Manguinhos%0D+%0D+Rio+de+Janeiro,+Brasil+21040-360&entry=gmail&source=g)  [Rio de Janeiro, Brasil 21040-360](https://maps.google.com/?q=Avenida+Brasil,+4365%0D+%0D+Manguinhos%0D+%0D+Rio+de+Janeiro,+Brasil+21040-360&entry=gmail&source=g)  Phone: 55-21-38659122  E-mail: [emilia.jalil@ini.fiocruz.br](mailto:emilia.jalil@ini.fiocruz.br) |
| M. Cristina Pimenta, PhD | Co-investigator | **Institution:** Ministry of Health - Departamento de Doenças de Condições Crônicas e IST  SRTVN Quadra 701, Lote D, Edifício PO700 – 5º andar, Brasília, DF Brasil 70719-040  Phone: [55-61-3315-2425](javascript:void(0))  E-mail: [cristina.pimenta@aids.gov.br](mailto:cristina.pimenta@aids.gov.br) |
| Thiago S. Torres, PhD | Co-investigator and technical advisor for pharmaceutical issues | **Institution:** Instituto Nacional de Infectologia Evandro Chagas - Fundação Oswaldo Cruz  [Avenida Brasil 4365](https://maps.google.com/?q=Avenida+Brasil+4365%0D+%0D+Rio+de+Janeiro,+Brasil&entry=gmail&source=g), Manguinhos  [Rio de Janeiro, Brasil](https://maps.google.com/?q=Avenida+Brasil+4365%0D+%0D+Rio+de+Janeiro,+Brasil&entry=gmail&source=g) 21040-360  Phone: 55-21-2270-7064  E-mail: [thiago.torres@ini.fiocruz.br](mailto:thiago.torres@ini.fiocruz.br) |
| Ronaldo Ismério Moreira, PhD | Statistician | **Institution:** Instituto Nacional de Infectologia Evandro Chagas - Fundação Oswaldo Cruz  [Avenida Brasil 4365](https://maps.google.com/?q=Avenida+Brasil+4365%0D+%0D+Rio+de+Janeiro,+Brasil&entry=gmail&source=g), Manguinhos  [Rio de Janeiro, Brasil](https://maps.google.com/?q=Avenida+Brasil+4365%0D+%0D+Rio+de+Janeiro,+Brasil&entry=gmail&source=g) 21040-360  Phone: 55-21-2270-7064  E-mail: [ronaldo.ismerio@ini.fiocruz.br](mailto:ronaldo.ismerio@ini.fiocruz.br) |
| Iuri Leite, PhD | Statistician | **Institution:** Escola Nacional de Saúde Pública - Fundação Oswaldo Cruz  [Avenida Brasil 4365](https://maps.google.com/?q=Avenida+Brasil+4365%0D+%0D+Rio+de+Janeiro,+Brasil&entry=gmail&source=g), Manguinhos  [Rio de Janeiro, Brasil](https://maps.google.com/?q=Avenida+Brasil+4365%0D+%0D+Rio+de+Janeiro,+Brasil&entry=gmail&source=g) 21040-360  Phone: 55-21-2270-7064  E-mail: [iuri.fiocruz@gmail.com](mailto:iuri.fiocruz@gmail.com) |
| Starley B. Shade, PhD, MPH | Statistician | **Institution**: Institute for Global Health Sciences - University of California San Francisco  San Francisco, USA  E-mail: Starley.Shade@ucsf.edu |
| Monica Derrico | Data management and quality control | **Institution:** Instituto Nacional de Infectologia Evandro Chagas - Fundação Oswaldo Cruz  [Avenida Brasil, 4365](https://maps.google.com/?q=Avenida+Brasil,+4365%0D+%0D+Manguinhos%0D+%0D+Rio+de+Janeiro,+Brasil+21040-360&entry=gmail&source=g), [Manguinhos](https://maps.google.com/?q=Avenida+Brasil,+4365%0D+%0D+Manguinhos%0D+%0D+Rio+de+Janeiro,+Brasil+21040-360&entry=gmail&source=g)  [Rio de Janeiro, Brasil 21040-360](https://maps.google.com/?q=Avenida+Brasil,+4365%0D+%0D+Manguinhos%0D+%0D+Rio+de+Janeiro,+Brasil+21040-360&entry=gmail&source=g)  Phone: 55-21-38659666  E-mail: [monica.derrico.imprep@gmail.com](mailto:monica.derrico.imprep@gmail.com) |
| Luana Monteiro Spindola Marins | Pharmacist | **Institution:** Instituto Nacional de Infectologia Evandro Chagas - Fundação Oswaldo Cruz  [Avenida Brasil, 4365](https://maps.google.com/?q=Avenida+Brasil,+4365%0D+%0D+Manguinhos%0D+%0D+Rio+de+Janeiro,+Brasil+21040-360&entry=gmail&source=g), [Manguinhos](https://maps.google.com/?q=Avenida+Brasil,+4365%0D+%0D+Manguinhos%0D+%0D+Rio+de+Janeiro,+Brasil+21040-360&entry=gmail&source=g)  [Rio de Janeiro, Brasil 21040-360](https://maps.google.com/?q=Avenida+Brasil,+4365%0D+%0D+Manguinhos%0D+%0D+Rio+de+Janeiro,+Brasil+21040-360&entry=gmail&source=g)  Phone: 55-21-38659666  E-mail: [luana.marins@ini.fiocruz.br](mailto:luana.marins@ini.fiocruz.br) |
| Sandro C. Nazer | INI/Fiocruz Laboratory Director | **Institution:** Instituto Nacional de Infectologia Evandro Chagas - Fundação Oswaldo Cruz  [Avenida Brasil, 4365](https://maps.google.com/?q=Avenida+Brasil,+4365%0D+%0D+Manguinhos%0D+%0D+Rio+de+Janeiro,+Brasil+21040-360&entry=gmail&source=g), [Manguinhos](https://maps.google.com/?q=Avenida+Brasil,+4365%0D+%0D+Manguinhos%0D+%0D+Rio+de+Janeiro,+Brasil+21040-360&entry=gmail&source=g)  [Rio de Janeiro, Brasil 21040-360](https://maps.google.com/?q=Avenida+Brasil,+4365%0D+%0D+Manguinhos%0D+%0D+Rio+de+Janeiro,+Brasil+21040-360&entry=gmail&source=g)  Phone: 55-21-38659666  E-mail: [sandro.nazer@ini.fiocruz.br](mailto:sandro.nazer@ini.fiocruz.br) |
| Marcos R. Benedetti, MA | Project Manager | **Institution:** Instituto Nacional de Infectologia Evandro Chagas - Fundação Oswaldo Cruz  [Avenida Brasil, 4365](https://maps.google.com/?q=Avenida+Brasil,+4365%0D+%0D+Manguinhos%0D+%0D+Rio+de+Janeiro,+Brasil+21040-360&entry=gmail&source=g), [Manguinhos](https://maps.google.com/?q=Avenida+Brasil,+4365%0D+%0D+Manguinhos%0D+%0D+Rio+de+Janeiro,+Brasil+21040-360&entry=gmail&source=g)  [Rio de Janeiro, Brasil 21040-360](https://maps.google.com/?q=Avenida+Brasil,+4365%0D+%0D+Manguinhos%0D+%0D+Rio+de+Janeiro,+Brasil+21040-360&entry=gmail&source=g)  Phone: 55-21-2270-7064  E-mail: [marcos.benedetti@ini.fiocruz.br](mailto:marcos.benedetti@ini.fiocruz.br) |
| Júlio Moreira | Community Engagement Coordinator | **Institution:**Grupo Arco-Íris de Cidadania LGBT  Rua da Carioca, 45, Rio de Janeiro, RJ, Brazil 20050-008  Phone: 55-21-2215-0844  E-mail: [jmoreira.imprep@gmail.com](mailto:jmoreira.imprep@gmail.com) |
| Keila Simpson | Community Engagement Coordinator | **Institution:**Associação Nacional de Travestis e Transexuais (ANTRA)  E-mail: atrasba@yahoo.com.br |
| Gabrielle O’Malley | Implementation Science Consultant | **Institution:** Department of Global Health - Schools of Medicine and Public Health - University of Washington  Seattle, Washington, USA  E-mail: [gabomalley@comcast.net](mailto:gabomalley@comcast.net) |
| Carolyn Yanavich | Laboratory Consultant | **Institution:** Instituto Nacional de Infectologia Evandro Chagas - Fundação Oswaldo Cruz  [Avenida Brasil, 4365](https://maps.google.com/?q=Avenida+Brasil,+4365%0D+%0D+Manguinhos%0D+%0D+Rio+de+Janeiro,+Brasil+21040-360&entry=gmail&source=g), [Manguinhos](https://maps.google.com/?q=Avenida+Brasil,+4365%0D+%0D+Manguinhos%0D+%0D+Rio+de+Janeiro,+Brasil+21040-360&entry=gmail&source=g)  [Rio de Janeiro, Brasil 21040-360](https://maps.google.com/?q=Avenida+Brasil,+4365%0D+%0D+Manguinhos%0D+%0D+Rio+de+Janeiro,+Brasil+21040-360&entry=gmail&source=g)  Phone: 55-21-2270-7064  E-mail: cmyanavich@gmail.com |

**Other Study Sites**

| **Name** | **Roles and Responsibilities** | **Contact Details** |
| --- | --- | --- |
| Alessandro Farias, MD | Site PI, Salvador/BA | **Institution**: CEDAP - Centro Especializado em Diagnóstico, Assistência e Pesquisa  Rua Comendador José Alves Ferreira, 240, Garcia, Salvador, BA Brasil 40100-010  Phone: 55-71-3116-6888  E-mail: [farias.alessandro@gmail.com](mailto:farias.alessandro@gmail.com) |
| Marcus V. Lacerda, MD, PhD | Site PI, Manaus/AM | **Institution**: Fundação de Medicina Tropical Heitor Vieira Dourado  Av. Pedro Teixeira, 25, Dom Pedro, Manaus, AM Brasil 69040-000  Phone: 55-92-2127-3555  E-mail: [marcuslacerda.br@gmail.com](mailto:marcuslacerda.br@gmail.com) |
| José Valdez Madruga, MD | Site PI, São Paulo/SP | **Institution**: Centro de Referência e Treinamento em DST/Aids de São Paulo  Rua Santa Cruz, 81 Vila Mariana, São Paulo, SP Brasil 04121-000  Phone: 55-11-5087-9999  E-mail: [josevaldezmadruga@gmail.com](mailto:josevaldezmadruga@gmail.com) |
| Josué N. de Lima, MD, PhD | Site PI, Campinas/SP | **Institution**: Centro de Referência em IST/Aids de Campinas (SMS/PMC)  Rua Regente Feijó 637 - Centro, Campinas, SP Brasil 13013-000  Phone: 55-19-3234-5000  E-mail: [josuenlima@hotmail.com](mailto:josuenlima@hotmail.com) |
| Ronaldo Zonta, MD | Site PI, Florianópolis/SC | **Institution**: CTA/Policlínica Centro  Av. Rio Branco, 90, Centro, Florianópolis, SC Brasil 88010-400  Phone: 55-48-3952-0100  E-mail: [ronaldozonta@gmail.com](mailto:ronaldozonta@gmail.com) |

1. Selected Implementation Science Constructs

| **Construct Domains and Definitions** |
| --- |
| **Theoretical Framework for Acceptability (TFA)^1^** |
| **Affective attitude**  How someone feels about the intervention |
| **Burden**  The perceived amount of effort that is required to participate in an intervention |
| **Ethicality**  The extent to which the intervention has good fit with an individual’s value system |
| **Intervention coherence**  The extent to which the participant understands the intervention, and how the intervention works |
| **Opportunity Cost/relative advantage**  The extent to which effort to use/deliver CAB LA PrEP is better than alternatives |
| **Perceived Effectiveness**  The extent to which the intervention is perceived to be likely to achieve its purpose |
| **Self-efficacy**  The participant’s confidence that they can perform the behavior(s) required to participate in the intervention |
| **Proctor Implementation Outcome ^2^** |
| **Feasibility**  The extent to which a new treatment, or an innovation, can be successfully used or carried out within a given agency or setting. (Drawing from Proctor, 2011) |
| **RE-AIM^3^** |
| **Reach**  The absolute number, proportion, and representativeness of individuals who are willing to participate in an intervention, and reasons why or why not |
| **Effectiveness**  The impact of an intervention on important individual outcomes, including potential negative effects, and broader impact including quality of life and economic outcomes; and variability across subgroups (generalizability or heterogeneity of effects). |
| **Implementation**  The fidelity to the various elements of an intervention’s key functions or components, including consistency of delivery as intended and the time and cost of the implementation. Importantly, it also includes adaptations made to interventions and implementation strategies and reasons for the above results. |
| **Maintenance** At the individual level: the long-term effects of a program on outcomes after a program is completed. The specific time frame for assessment of maintenance or sustainment varies across projects. |

## Exclusion Criteria

- One or more reactive or positive HIV test result at enrollment visit, even if HIV infection is not confirmed.
- Currently participating in interventional trial of PrEP agents, HIV vaccine trial or experimental medication.
- Positive pregnancy test, breastfeeding, or intention to become pregnant at enrolment (for transgender men).
- Prior participation in studies with Cabotegravir.
- A history or presence of allergy to the study drug components.
- Past participation in HIV vaccine trial. An exception will be made for participants that can provide documentation of receipt of placebo (not active arm).
- Plan to relocate out of the area during the study period.
- Surgically placed or injected buttock implants or fillers, per self-report.
- Has a dermatological/inflammatory skin condition overlying the buttock region which in the opinion of the study investigator, in consultation with the Clinical Study Coordination (CSC), may interfere with interpretation of injection site reactions.
- Active or planned use of contraindicated co-administered for which significant decreases in Cabotegravir plasma concentrations may occur due uridine diphosphate glucuronosyltransferase (UGT1A1):
  - - Anticonvulsants: Carbamazepine, Oxcarbazepine, Phenobarbital, Phenytoin
    - Antimycobacterials: Rifampin, Rifapentine
- **Any prohibited medications that decrease CAB concentrations should be discontinued for a minimum of four weeks or a minimum of three half-lives (whichever is longer) prior to the first dose.*
- Current or anticipated need for chronic systemic anticoagulation or a history of known or suspected bleeding disorder, including a history of prolonged bleeding.
- History of severe hepatic impairment (including but not limited to a history of liver failure or hospitalization for liver disease, a history of hepatocellular carcinoma or near liver transplant).
- Individuals with advanced Hepatitis C.
- Other medical conditions that, in the opinion of the study investigator, would interfere with the conduction of the study (e.g., provided by self-report, or found upon medical).

## Objectives and Endpoints

**Primary Objectives**

Implementation objective**:**

- Facilitators of integration of CAB-LA into existing PrEP services
- Barriers to integration of CAB-LA into PrEP existing PrEP services

Clinical objective:

- Incident HIV infection (Primary outcome)

**Secondary Objectives**

- PrEP choice
- PrEP initiation
- Adherence to injection visits
- Switch from CAB-LA to oral PrEP
- Discontinuation of CAB-LA PrEP
- Reason(s) for PrEP choice
- Facilitators of coming to injection visits
- Barriers to coming to injection visits
- Reason(s) for switching PrEP agent
- Reason(s) for discontinuation of CAB-LA PrEP
- PrEP knowledge scale score
- Acceptability of mHealth intervention
- Feasibility of mHealth intervention
- Acceptability of WhatsApp appointment reminders
- Acceptability of HIV testing strategy
- Weight
- Injection site pain
- Severe adverse events
- Resistance to CAB-LA or oral TDF/FTC PrEP
- HIV positivity by diagnostic test (3^rd^ Gen RT, 4^th^ Gen RT, GeneXpert VL, HIVST)
- HIV viral load at time of diagnosis.
- CAB LA drug concentration at time of diagnosis.
- Weeks on ART before achieving viral suppression.

**Tertiary Objectives**

- Prevalence of STI infection
- Incidence of STI infection
- Feasibility of self-collected samples for STI testing (rectal/urine/oropharyngeal)
- STI tests completed
- STI test results
- Acceptability of self-collected samples for STI testing (rectal/urine/oropharyngeal)

1. **Interim Analysis**

We will conduct interim analysis of HIV incidence in participants who select CAB-LA for HIV prevention.

**Intervention Sample:** Participants who select CAB-LA PrEP

**Comparison Sample:** Individuals who initiate daily PrEP during the enrollment period within clinics in the Brazilian Health System

**Censoring Time:** Study time is censored at the enrollment period (~12 months after initiation of enrollment in each site).

**Endpoint:** Among individuals who test positive on HIV rapid test, midpoint between last negative HIV rapid test and first HIV positive rapid test

**Analysis:** Our interim analysis will employ an intent-to-treat (ITT) approach and compare HIV incidence (and 95% CI) in CAB-LA cohort to HIV incidence (and 95% CI) among individuals on daily PrEP within the Brazilian. We will estimate the rate of HIV incidence with 95% confidence interval in each group using Kaplan-Maier statistics. We will employ a Cox Proportional Hazard model to assess the relative hazard (Hazard Ratio with 95% confidence interval) between the two groups.

**Sample Size Considerations:** We anticipate enrollment of 200 individuals into CAB-LA PrEP over 12 months in each participating clinic (total of 1,200). At the end of the enrollment period, we anticipate an average follow-up of 0.5 years per person with at least 85% retention (N=510 person-years of follow-up). If our comparison group is of similar size, then we would need to observe at least 7 incident cases to observe statistically significant differences between CAB-LA and oral PrEP. If our comparison group is three times larger, then we would need to observe 13 or more incident cases to observe a statistically significant difference between groups. Similarly, if we observe 1 incident case, then we will be able to estimate and incidence of 1.96 per 100 person years with a 95% confidence interval of 0.03, 10.91 per 100 person-years. If our comparison group is of similar size, then we would need to observe at least 8 incident cases and if our comparison group is three times greater, then we would need to observe at least 19 incident cases to observe a statistically significant difference between groups.
